# Distinct proteome pathology of circulating microparticles in systemic lupus erythematosus

**DOI:** 10.1186/s12014-017-9159-8

**Published:** 2017-06-21

**Authors:** Ole Østergaard, Christoffer Tandrup Nielsen, Julia T. Tanassi, Line V. Iversen, Søren Jacobsen, Niels H. H. Heegaard

**Affiliations:** 1 Department of Autoimmunology and Biomarkers, Statens Serum Institut, Copenhagen, Denmark; 2 Copenhagen Lupus and Vasculitis Clinic, Centre for Rheumatology and Spine Diseases, Rigshospitalet, Copenhagen University Hospital, Copenhagen, Denmark; 3 Department of Dermatology, Aarhus University Hospital, Aarhus, Denmark; 4 Department of Clinical Biochemistry and Pharmacology, Odense University Hospital, University of Southern Denmark, Odense, Denmark

**Keywords:** Circulating microparticles, Systemic autoimmunity, Systemic lupus erythematosus, Biomarkers, Proteome

## Abstract

**Background:**

The pathogenesis of systemic lupus erythematosus (SLE) is poorly understood but has been linked to defective clearance of subcellular particulate material from the circulation. This study investigates the origin, formation, and specificity of circulating microparticles (MPs) in patients with SLE based on comprehensive MP proteome profiling using patients with systemic sclerosis (SSc) and healthy donors (HC) as controls.

**Methods:**

We purified MPs from platelet-poor plasma using differential centrifugation of samples from SLE (n = 45), SSc (n = 38), and two sets of HC (n = 35, n = 25). MP proteins were identified and quantitated after trypsin digestion by liquid chromatography-tandem mass spectrometry. The abundance of specific proteins was compared between the groups using univariate statistics and false discovery rate correction for multiple comparisons. Specific proteins and protein ratios were explored for diagnostic and disease activity information using receiver-operating characteristic curves and by analysis of correlations of protein abundance with disease activity scores.

**Results:**

We identify and quantitate more than 1000 MP proteins and show that a subpopulation of SLE-MPs (which we propose to call luposomes) are highly specific for SLE, i.e. not found in MP preparations from HC or patients with another autoimmune, systemic disease, SSc. In SLE-MPs platelet proteins and mitochondrial proteins are significantly diminished, cytoskeletal proteins deranged, and glycolytic enzymes and apoptotic proteins significantly increased.

**Conclusions:**

Normal MPs are efficiently removed in SLE, but aberrant MPs, derived from non-lymphoid leukocytes, are less efficiently removed and abundantly produced leading to an altered MP proteome in SLE. The data suggest that an abnormal generation of MPs may partake in the pathology of SLE and that new diagnostic, monitoring, and treatment strategies targeting these processes may be advantageous.

**Electronic supplementary material:**

The online version of this article (doi:10.1186/s12014-017-9159-8) contains supplementary material, which is available to authorized users.

## Background

Cells and tissues release subcellular membrane-enclosed fragments (extracellular vesicles). These vesicles are heterogeneous and include cell membrane-derived microparticles (MPs), exosomes from multivesicular bodies, and larger membrane particles from apoptotic and necroptotic cells and from cells undergoing other types of cell death [1]. Extracellular vesicles carry out physiological functions [2] and potentially are easily accessible markers of specific pathology [3].

Systemic lupus erythematosus (SLE) is an autoimmune disease characterized by chronic and fluctuating inflammation, circulating autoantibodies, and immune complex-mediated tissue damage [4]. Clearance defects have long been implicated in the SLE pathogenesis [5, 6] and experimental data support that waste generation and removal are associated with pro-inflammatory mechanisms in SLE [7–12]. It is, however, unsettled if the common human SLE phenotype primarily involves clearance defects, i.e., if the production and release of MPs to the circulation is normal while their clearance is compromised or if there is an aberrant production of MPs overwhelming or bypassing normal removal mechanisms leading to systemic accumulation of autoinflammatory and autoimmunogenic material. Under normal conditions, circulating MPs derive predominantly from platelets, leukocytes, and endothelial cells [13] and may be extensively profiled by mass spectrometry [14, 15]. Abnormal MPs [16–18] with anomalous protein profiles [19] were shown to circulate in SLE.

To understand the genesis and function of abnormal MPs in SLE we here perform an unbiased, comprehensive, and detailed characterization of MP-proteomes in SLE and controls using large sample sets and high-resolution, quantitative protein analysis. We identify and quantitate about 1100 different proteins in MPs from SLE, systemic sclerosis (SSc), and healthy controls. Data indicate that the clearance of normal MPs in SLE is increased but that abnormal, SLE-specific MPs, which we name luposomes, derived from non-lymphoid leukocytes, are increased in SLE and bear hallmarks of apoptosis and metabolic stress.

## Methods

### Patients and controls

Forty-five SLE patients (42 women, 3 men) were included (Additional file 1: Table S1). Of these 44 fulfilled at least four of the 1997 ACR criteria for SLE while one had primary antiphospholipid syndrome (APS) [20, 21]. Twelve patients had SLE with secondary APS. Overall, 15 of 44 (34%) had serological and/or clinical evidence of active disease (SLEDAI > 4) and 30% had active or prior lupus nephritis. The age and gender matched controls for the SLE cohort consisted of 35 healthy individuals (31 women, 4 men), median age 45 years (range 24–71 years). Additionally, 38 SSc patients were included, all Caucasians (29 women and 9 men), median age 57 years (range 35–74), median disease duration 12 years (range 0–53 years). Patients fulfilled the SSc criteria [22], 18 with limited cutaneous SSc and 20 with diffuse cutaneous SSc [23, 24]. The SSc age and gender matched controls consisted of 25 healthy individuals (6 male, 19 female), median age 45 years (range 31–62). The study was approved by the Scientific Ethical Committees for the Capital Region, Denmark (approval numbers H-B-2007-130 and H-B-2008-131) and carried out according to the principles of the Declaration of Helsinki. All participants were included after giving written informed consent. Disease activity was scored with the SELENA version of the SLE Disease Activity Index (SLEDAI) [25]. Standard hematology parameters were acquired on a Sysmex XN-9000 hematology analyzer (Sysmex Corporation).

### Sampling and preparation of platelet-poor plasma

Platelet-poor plasma (PPP) was isolated using a standardized protocol [17]. Briefly, venous blood was drawn into citrate tubes (Vacuette sodium citrate 3.8%, Greiner Bio-One, Kremsmünster, Austria). Immediately after blood collection, cells were removed by centrifugation (1800 g, 10 min, 21 °C) followed by centrifugation at 3000 g, 10 min, 21 °C to remove platelets. Samples were divided into 1000 μL aliquots, snap-frozen in liquid nitrogen, and stored at −80 °C.

### Microparticle flow cytometry

Flow cytometry using gating based on size-beads was performed exactly as previously described [17].

### Isolation of microparticles and in-solution protein digestion

MPs were isolated from 1 mL PPP by centrifugation at 18,890×*g*, 30 min at room temperature as previously described [15]. After the last wash the MP preparations (50 μL) were precipitated by trichloroacetic acid/acetone. Proteins were resolubilized in 25 μL 8 M urea, 50 mM NH_4_HCO_3_ and digested 3 h at room temperature using endo-Lys C (0.5 μg/50 μL; Waco Pure Chemical Industries Ltd, Osaka, Japan) before dilution to 2 M urea using 50 mM NH_4_HCO_3_ and continued digestion overnight at room temperature in the presence of 1 μg/50 μL sequencing grade modified trypsin (Promega, Madison, USA). Samples were then frozen until analysis by LC–MS/MS.

### Nano-LC-tandem mass spectrometry

The digested samples were thawed, mixed on a whirlmixer, acidified (5% (v/v) formic acid, final concentration), and centrifuged (12,000×*g*/2 min) to remove insoluble material. 10 μL was desalted on pre-equilibrated homemade StageTip columns containing C18 Empore Disks (3 M, Minneapolis, MN) [26] by washing with 20 μL 5% formic acid followed by elution of the peptides with 20 μL 50% methanol, 5% formic acid into a 0.65 mL Eppendorf tube. The peptides were vacuum concentrated until almost complete dryness and re-dissolved in 20 μL 5% formic acid. Samples were then analyzed in random order within each sample cohort (SLE and HCs and SSc and HCs). LC–MS/MS was performed by loading 5 μL of the peptides at 200 nL/min on an Acclaim PepMap100 C18 precolumn (100 μm × 2 cm, 5 μm particle size, 100 Å, Thermo Fischer Scientific) in line with an EASY-spray PepMap100 C18 analytical column with integrated emitter (75 μm inner diameter, 150 mm long, 3 μm particle size, 100 Å, Thermo Fisher Scientific). Peptides were eluted by a 90-min gradient controlled by an Easy-nLC II pump (Thermo Fisher Scientific) into an LTQ Orbitrap XL mass spectrometer (Thermo Fisher Scientific) equipped with an EASY-spray nano-electrospray source (Proxeon, Odense, Denmark). Mobile phases were: solvent A (2% (v/v) acetonitrile, 0.1% (v/v) formic acid) and solvent B (95% (v/v) acetonitrile, 0.1% (v/v) formic acid). The gradient went from 0 to 45% solvent B in 80 min, followed by 10 min with 100% solvent B. The column was then re-equilibrated with solvent A. Full scan spectra (300–1800 mass/charge [m/z]) in the Orbitrap with 60,000 resolution at 400 m/z and MS/MS data were recorded in parallel in data-dependent mode, fragmenting the 5 most abundant ions (charge state +2 or higher) by collision-induced dissociation in the LTQ ion trap at 35% collision energy. MS/MS spectra were recorded using dynamic exclusion (20 s) to minimize repeated fragmentation of the same peptides.

### Data analysis

Recorded.raw files were analyzed using MaxQuant version 1.2.2.5 (SLE samples) and version 1.1.1.36 (SSc samples) for label-free peptide quantitation by MS1-intensity and for protein identification using the Andromeda search engine. Settings were: FASTA-file: ipi.HUMAN.v3.68.fasta (UniProt__3AUP000007640.fasta was used for Epstein-Barr virus proteins), fixed modifications: none, variable modifications: oxidation (M), acetyl (protein N-terminal), Peptide FDR 1%, Protein FDR 1%, match between runs 2 min, Keep low-scoring version of identified peptides: on; all other settings were left at their default. For each identified protein we used the summed MS1-peptide intensities calculated by MaxQuant (in short “the protein intensities”) for the respective comparisons of protein expression between groups. Differences in MP numbers across samples were accounted for by using the protein intensity of the high abundance MP protein β-actin as a surrogate parameter for the number of isolated vesicles from each sample and normalizing protein intensities of all proteins by the β-actin intensity as follows [15]: The normalized intensity of a given protein is found by multiplying the value with the ratio between the average β-actin intensity for all samples divided by the β-actin intensity in the specific sample. To compare relative abundances of proteins within individual samples we used IBAQ (intensity-based absolute quantification) values [27]. Inadvertently, two MaxQuant software versions were used to extract protein identification and quantitation data in the two sample cohorts (SLE & controls and SSc & controls), respectively. We therefore do not compare across sample cohorts but only within sample cohorts. Also, in a subset of samples we performed the data extraction using both versions of the software and observed linear (R^2^ > 0.99) correlations of intensity values and that missing identifications only occurred among low abundance proteins (data not shown). Data files are provided as Additional files 2 and 3.

### Statistical methods

For comparison of specific proteins between groups we used Mann–Whitney two-tailed tests and corrected *p* values by the false discovery rate [28] using *q* < 0.05 as the significance level. Only proteins with *q*-values below 0.05 and SLE/HC mean ratios >1.25 or <0.75 were considered differentially abundant. For correlations of disease activity scores (SLEDAI) with protein intensity values we used Spearman’s rank correlation. Calculations and graphs were performed with Prism v. 6.0 (GraphPad Software, Inc., La Jolla, CA). Clustering heat maps were made using Genesis (v. 1.7.6, Institute for Genomics and Bioinformatics, Graz University of Technology).

## Results

### Cytoskeletal changes in SLE-MPs

**Fig. 1:**
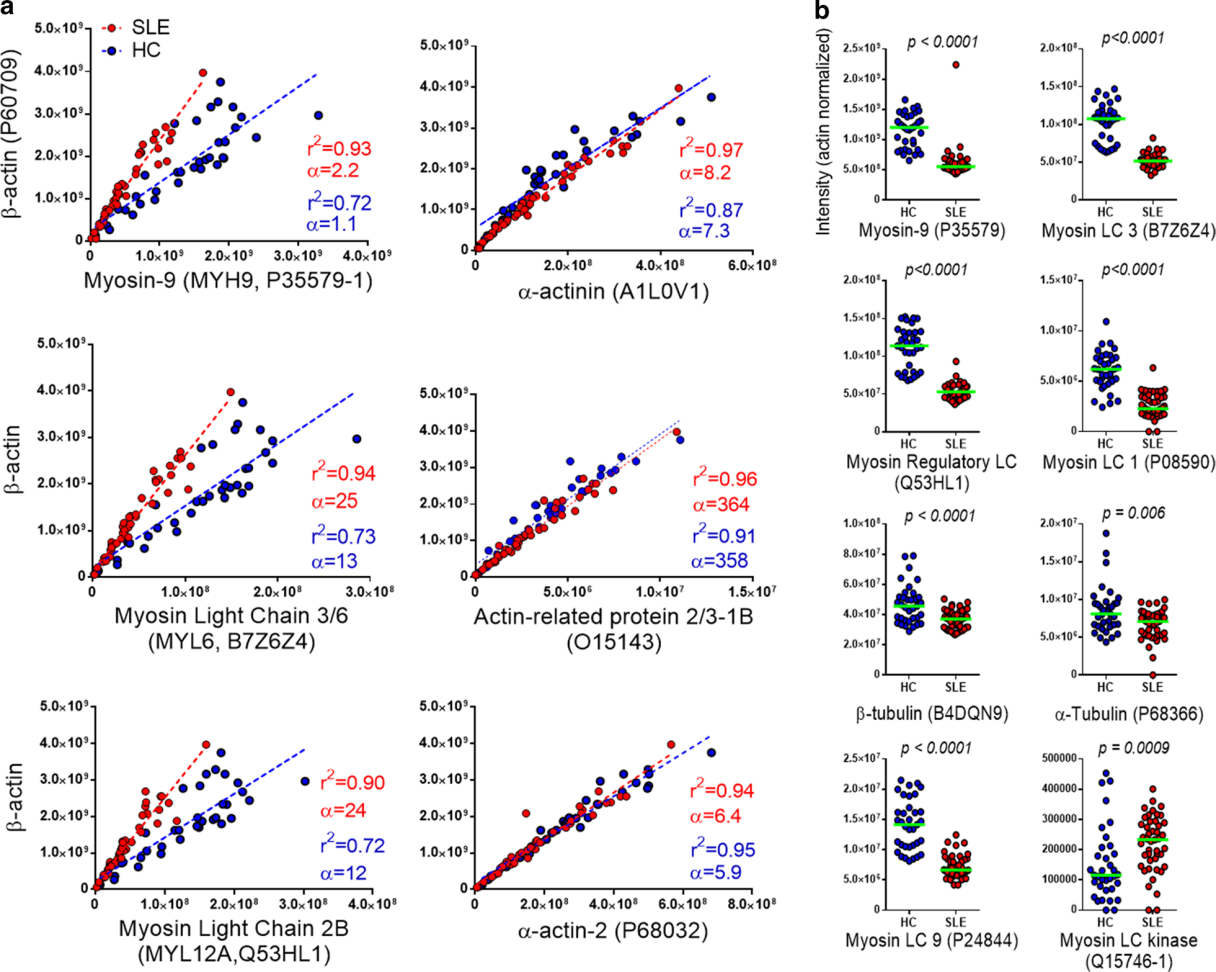
Specifically altered myosin:actin ratio in SLE-MPs. **a** Individual, raw β-actin intensity values as a function of myosin and other cytoskeletal protein intensities. Correlation coefficients (r^2^) and slopes (α) are given for the regression lines of the SLE-MPs (*red*) and the HC-MPs (HC, *blue*). In the SLE-MPs the slope of the *lines* for the myosin heavy chain (MYH9) and the myosin light chains are approximately twice the slopes found for the HC-MPs while no differences are observed for the α-actinin, α-actin-2, and actin-related protein. UniProt identifiers are included on the X-axes. **b** Intensity values of actin-normalized myosins, tubulins, and myosin light chain kinase in the SLE-MP and HC-MP cohort (*red* and *blue symbols*, respectively), medians (*horizontal lines*) and *p* values are indicated (Mann–Whitney, two-tailed)

Both β-actin and myosin-9 intensities correlated linearly with MP concentrations determined by flow cytometry (data not shown), but while the average raw intensity value of myosin-9 (*MYH9*) in the HC group was 2.7 times higher (*p* < 0.0001) than in the SLE group, the β-actin (*ACTB*) value was only 1.4 times higher (*p* = 0.014). Unlike β-actin myosin-9 in MPs is unaffected by snap-freezing [29] but as it was here consistently greatly decreased in SLE-MPs it was not suitable for normalization. The slightly lower β-actin values in SLE-MPs probably reflect the overall tendency to lower total MP numbers in SLE samples [17]. β-Actin values were therefore used to normalize all protein intensity data. Plots of β-actin versus myosin-9 and other cytoskeletal proteins (Fig. 1; Additional file 1: Fig. S1) indicated specifically altered ratios between myosins and β-actin in SLE-MPs. The consistent differences in the slopes of the regression lines (highly significant difference for *MYL6* but not significant for *MYH9* and *MYL12A*) suggested that about half the myosin *per* β-actin is present in SLE-MPs as compared with HC-MPs (Fig. 1a, left column). Myosin light and heavy chains ranked among the most significantly altered proteins in SLE-MPs [3 out of 5 proteins with *q* < 1 × 10^−9^ were myosins (*MYL12A, MYL6, MYH9*)] (Fig. 1b) and were unaltered in the SSc–HC MP data set. Microtubule proteins (α and β tubulins) were also decreased in SLE-MPs (Fig. 1b). In contrast, myosin light chain kinase (*MLCK*) was highly significantly increased (*q* = 0.0027) (Fig. 1b) but not in SSc-MPs. Other cytoskeletal proteins including vimentins, desmins, and nuclear lamins were not identified, and cytokeratins (24 different types identified) were not different between SLE and HC MPs. Unlike myosins, most other actin-binding proteins (Additional file 1: Fig. S2), except for decreased α-filamin (data not shown), were significantly increased in SLE-MPs.

### Proteomic profiles of circulating MPs

**Fig. 2:**
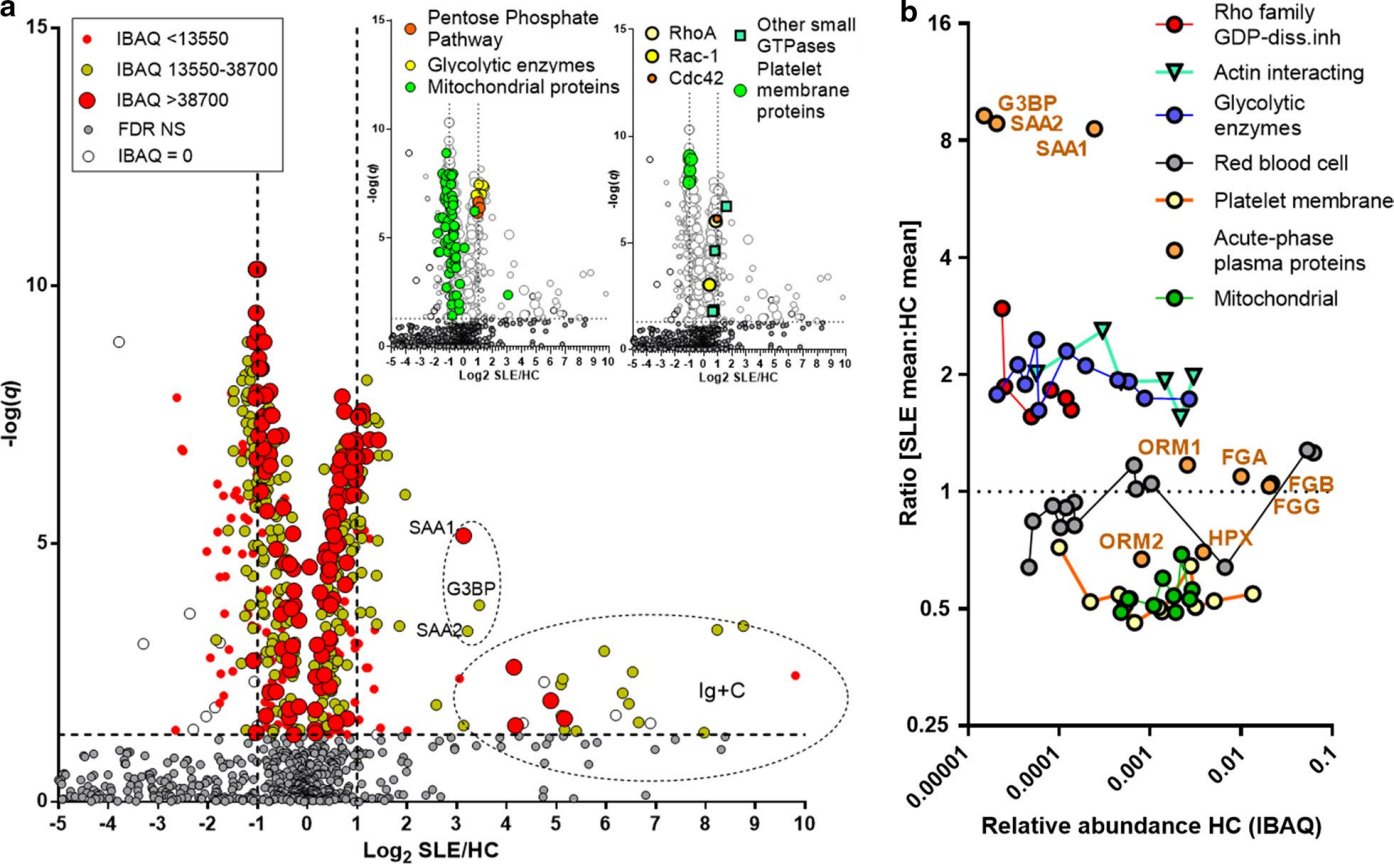
Comparison of the MP proteomes in SLE and HC. **a** Fold-change, abundance, and statistical difference of all proteins (n = 1098) identified in the SLE-MP versus HC-MP cohort. Protein abundances for all proteins (n = 505) with *q* < 0.05 are divided into 3 ranges (between 13,550 and 38,700 and values either below or above this range. Significant entries (n = 15) in the SLE group which have zero median IBAQ values (intensity-based absolute quantification analysis) are included as *open symbols*. X-axis is the SLE/HC average intensity (LFQ) value ratio depicted as Log_2_(SLE/HC). Y-axis is −log(*q*). *Vertical lines* mark twice up- or downregulation, while values above the *horizontal line* all are below *q* = 0.05, i.e. are statistically significant. Areas of the *plot* containing the serum amyloid A (SAA1, SAA2) and galectin-3 binding (G3BP) proteins and a number of immunoglobulins and complement proteins (Ig + C) are indicated. The *two insets* show the position in the plots of 4 chosen categories of proteins: platelet membrane and mitochondrial proteins which are highly significantly decreased in SLE-MPs and glycolytic and pentose pathway enzymes and small GTPases and their regulators that are highly upregulated in SLE-MPs. **b** Relative protein abundance based on the abundance ranking in SLE samples) and SLE/HC ratios (log(2) Y-axis) for chosen categories of proteins. Acute phase proteins and galectin-3 binding protein (G3BP) have been labelled: SAA1, SAA2, serum amyloid A protein, isoform 1 and 2, respectively; ORM, orosomucoid isoforms; HPX, hemopexin; FGA, FGB, FGG, fibrinogen α, β, and γ chains, respectively

In total, 1098 unique proteins were identified in the SLE–HC samples. Of these, 312 were present in all samples and 743 were present in at least 80%. In the SSc–HC samples, 1029 proteins were identified with 401 proteins present in all samples and 715 in at least 80%. The volcano plot (Fig. 2a) depicts the distribution of proteins according to abundance, statistical significance (*q*-values) and SLE/HC abundance ratio. Data show, in accordance with flow cytometry [30], that SLE-MPs have a heavy load of immunoglobulins and complement proteins (encircled Ig + C area) as well as large increases in galectin-3 binding protein (G3BP) and serum amyloid A (SAA) (small encircled area). Acute-phase proteins showed a dichotomous behavior—serum amyloid A1 and A2 proteins were highly upregulated in SLE-MPs while orosomucoids, hemopexin, and fibrinogens were unchanged (Fig. 2b). In contrast, acute-phase proteins were more consistently elevated in the SSc-MPs (Additional file 1: Fig. S3). C-reactive protein was detected in 50% of the SSc samples but was not found in any SLE sample.

Specific protein categories (inserts Fig. 2a) such as mitochondrial proteins and platelet membrane proteins were decreased, and pentose phosphate shunt and glycolytic enzymes and several small GTPases were highly increased in SLE-MPs but not in the SSc-MPs (data not shown). Most SLE/HC protein ratios were consistent within protein categories, *e.g.* decreased for platelet proteins, unaltered for erythrocyte membrane proteins, and increased for glycolytic enzymes and did not correlate with the abundance of the individual proteins (Fig. 2b).

**Fig. 3:**
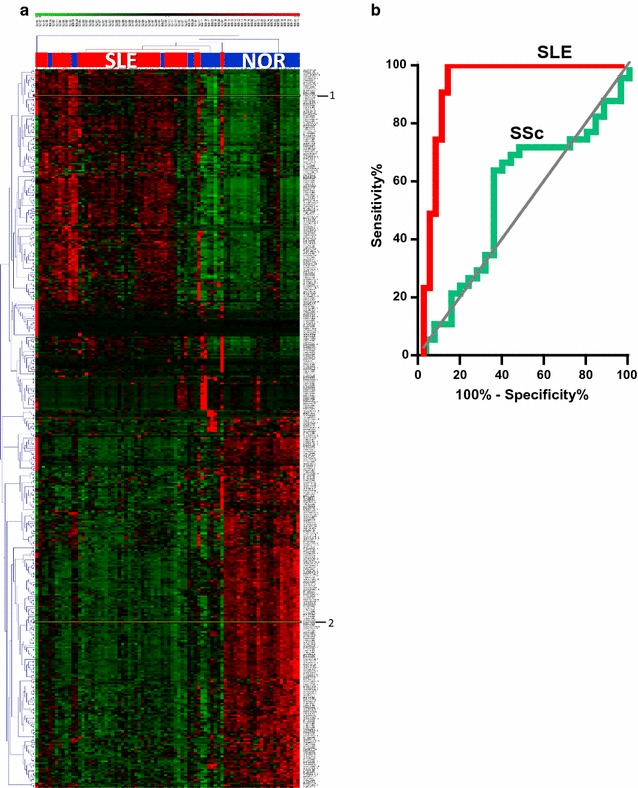
MP proteome profiling distinguishes SLE-MPs from HC-MPs. **a** Hierarchical clustering of SLE samples (*red color above heat map*) and HC samples (*blue color*) based on MP protein intensity data for a selection of 422 proteins that are significantly (*q* < 0.05) different between SLE and HC and with SLE/HC ratios ≥1.25 or ≤0.75. Heat map color code is based on the tiered *color bar* at the *top* of the plot going from 3 standard deviations below (*green*) to 3 standard deviations (*red*) above the mean value. **b** Receiver operating characteristics (ROC) curves for the ratio of α-enolase/cytochrome b-c1 complex subunit 2 (mitochondrial) (proteins marked with 1 and 2, respectively, in **a**) in the SSc–HC sample set (*green*, AUC = 0.54) and the SLE–HC sample set (*red*, AUC = 0.93)

Specific immunoglobulin entries show highly variable expression in the individual SLE samples. We therefore simplified the data by removing about 200 entries corresponding to immunoglobulin and complement proteins (as well as unknown, hypothetical, and non-human entries). In this data set, 240 proteins were significantly (*q* < 0.05) decreased (SLE/HC ≤ 0.75) and 182 were increased (SLE/HC ≥ 1.25). The largest group of decreased proteins were mitochondrial. Thus, 64 significantly decreased mitochondrial proteins together with 19 platelet/integrin proteins constituted 35% of all decreased proteins. Heat shock proteins, GTPases, and glycolytic enzymes represented about 15% of all increased proteins. Hierarchical clustering of the selected group of 422 significant proteins almost completely separated SLE from HC samples (Fig. 3a). This was also accomplished using all 930 proteins remaining after removing immunoglobulins, complement proteins, hypothetical and unknown proteins and *Bos taurus* proteins. Thus, a total of 40 out of 45 SLE samples clustered together only interspersed with 3 HC samples (Additional file 1: Fig. S4). To illustrate the potential for new diagnostic tools in the dataset we randomly picked a glycolytic enzyme (α-enolase, marked 1 in Fig. 3a) and a decreased mitochondrial protein (cytochrome bc1, marked 2) for a disease-specific index. This index yielded a discriminatory power of 0.93 for SLE versus HC (area under the ROC curve) (Fig. 3b). In contrast, for SSc versus HC (green, Fig. 3b), the area under the ROC curve was only 0.54, which is close to the equivalent of random chance. To fully show the applicability of this index it should be validated in independent samples.

**Fig. 4:**
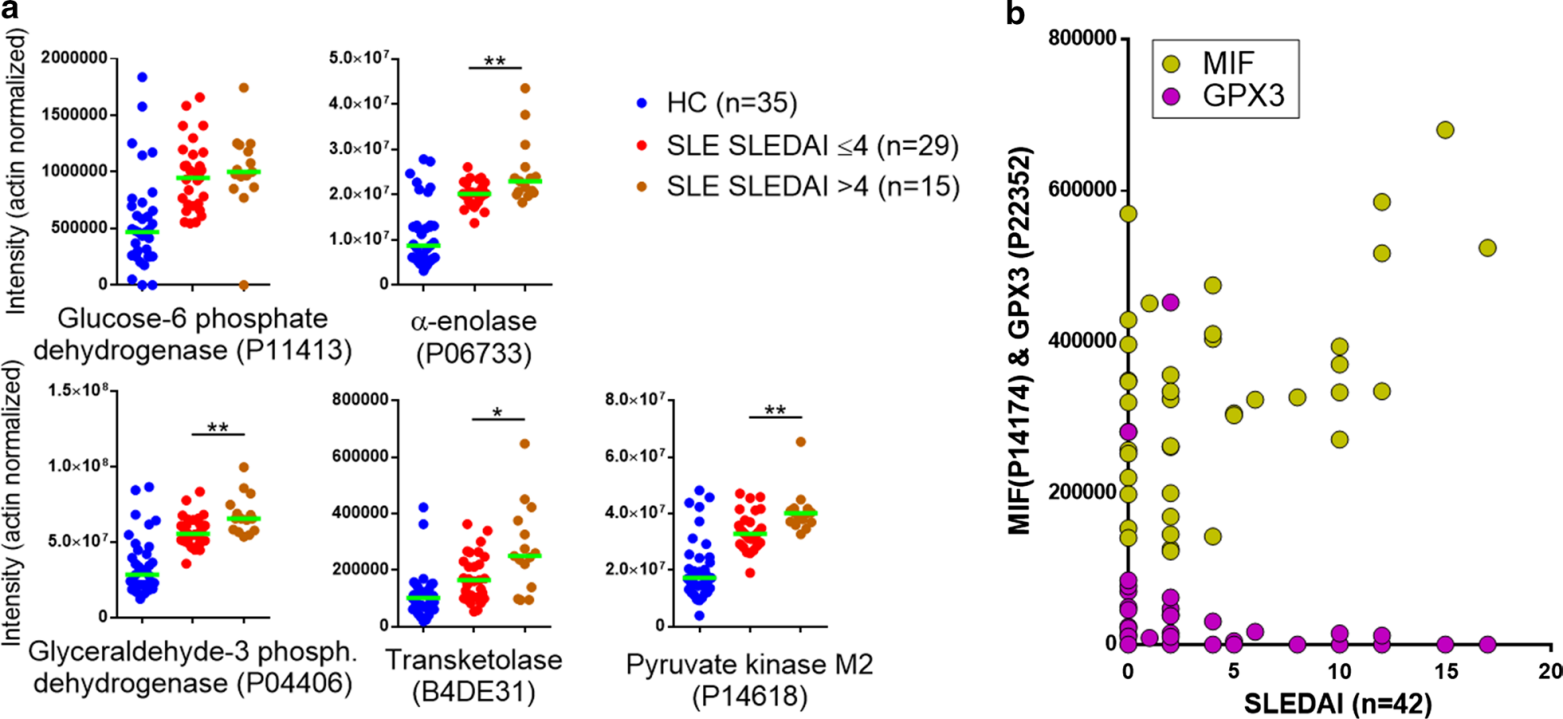
SLE-MP proteins and correlations with SLE disease activity indices (SLEDAI). **a** Levels of glycolytic enzymes in SLE samples from patients divided into two groups based on SLEDAI scores ≤4 (*red symbols*, low disease activity, n = 29) or above 4 (*brown symbols*, high disease activity, n = 15). Levels in the HC group are also shown. Except for a non-significant difference for glucose-6-phosphate dehydrogenase the glycolytic enzymes were increased significantly [*p < 0.05; **p < 0.01 (Mann–Whitney, two-tailed)] in the group with SLEDAI scores >4. **b** Correlation between protein intensities and SLEDAI scores for macrophage migration inhibitory factor (MIF, Spearman r = 0.37, *p* = 0.0156) and glutathione peroxidase 3 (GPX3, Spearman r = −0.52, *p* = 0.0005). Two SLE samples with zero MIF were removed from the sample set

Regarding correlations with a validated SLE disease activity index (SLEDAI), the anti-oxidant glutathione peroxidase 3 (*GPX3,* strongly decreased, *q* = 2.2 × 10^−7^) inversely correlated (*p* = 0.0005, Spearman rho = −0.52) with SLEDAI scores (Fig. 4b). Further, the glycolytic enzymes were most highly increased in samples from patients with the highest SLEDAI scores (i.e., >4, n = 15/44) (Fig. 4a). Also, there was a correlation (*p* = 0.02, rho = 0.37) between SLEDAI and macrophage migration inhibitory factor (*MIF*) (Fig. 4b). No significant correlations with active nephritis at time of sampling (n = 10) remained after correction for multiple comparisons.

### Contributions from normal MPs

In SLE-MPs the abundance of platelet membrane proteins was markedly decreased (by about 75%) (Fig. 2) while being unaltered in the SSc-MPs. Reduced numbers of platelet-derived CD42a (GP9)-positive MPs (PMPs) were previously noted in flow cytometry of MPs from SLE cases [17]. In the present sample set, despite normal counts (platelet mean ± SD = 242 ± 79 × 10^9^/L), the average number of PMPs in the SLE samples as determined by flow cytometry was decreased by 60% (data not shown), corresponding roughly to the proteomics estimate. PMP numbers correlated with the MS-determined abundance of GP9 (*p* = 0.0012, rho = 0.41) in the SLE–HC sample set and with the platelet counts (*p* = 0.03, rho = 0.33). Thus, the circulating MPs in SLE contain reduced numbers of PMPs despite normal platelet levels. T-lymphocyte membrane markers such as CD4, CD8, CD3 and traditional B cell markers, including e.g. CD19, CD20, CD38 and MHC class II molecules were not observed in any samples.

Red blood cell markers showed no significant differences between SLE or SSc and HC MPs (Fig. 2b). This was also the case for a number of identified CD molecules (examples in Additional file 1: Fig. S5) including CD45 (common leukocyte antigen), CD11b and CD18 (leukocyte integrin), CD14 (monocyte/macrophages, dendritic cells), CD16B (FcγRIIIb), and CD99 (leukocytes). Other integrins, CD49 and CD49B (platelet membrane glycoprotein Ia), integrin β_6_, fibrinogen receptor β subunit (VLA-4), as well as P-selectin (CD62P), CD36 (platelet glycoprotein IIIb), CD31 (PECAM-1, platelets, leukocytes, endothelial cells), and CD226 (NK cells, platelets, monocytes, T-cell subsets), were all strongly decreased in SLE-MPs (*q* < 0.0001). This was also the case for CD47/MER6 and for CD107/LAMP-1, an abundant lysosomal-associated glycoprotein. In contrast, cathepsin D, the principal lysosomal aspartic protease, was not significantly changed. Finally, CD9 was significantly decreased in SLE-MPs (Additional file 1: Fig. S5). CD9 is present in many types of extracellular vesicles together with CD63 and CD81 [31]. The latter two proteins were not detected here. Other decreased membrane-associated proteins in SLE-MPs were flotillins 1 and 2, which are often considered exosomal markers but which are also found in other extracellular vesicles [32] (Additional file 1: Fig. S6). The ER protein GP96 (endoplasmin, *HSP90B1*) that was recently nominated as a marker of large extracellular vesicles [32] was present at equal levels in SLE- and HC-MPs (data not shown).

### SLE-MPs and markers of apoptosis and clearance

**Fig. 5:**
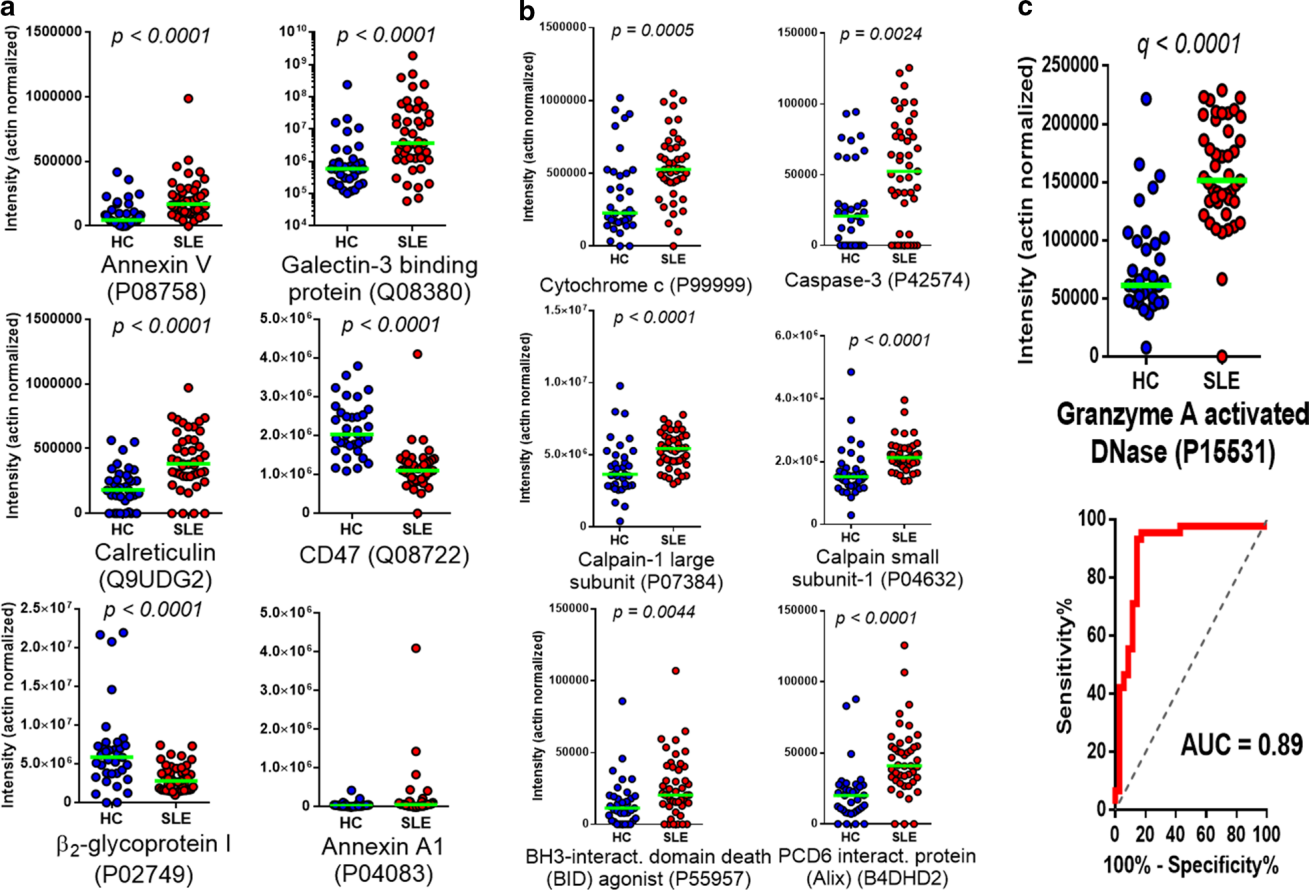
Increased levels of clearance proteins and proteins related to apoptosis in SLE-MPs. Intensities of specific clearance- (**a**) and apoptosis-related (**b**) proteins in HC (*blue symbols*) versus SLE-MPs (*red symbols*). Except for β_2_-glycoprotein 1 and CD47 (both significantly decreased) and annexin A1 (no difference), all proteins were significantly increased (Mann–Whitney, two-tailed) in the SLE-MP samples. Univariate *p* values and UniProt identifiers are indicated. Horizontal lines mark medians. **c** Levels of significantly increased granzyme A activated DNase/nm23-H1 and resulting ROC curve for SLE versus HC samples

While annexin V (*ANXA5*) was significantly increased in SLE-MPs, annexin A1 displayed no difference (Fig. 5a). Another apoptosis-related molecule, calreticulin (*CRTC*) was selectively increased in SLE-MP (about twofold, *q* < 0.0001). Calreticulin is externalized upon apoptosis [33]. The calcium-independent phosphatidylserine-binding proteins lactadherin and growth arrest-specific protein 6 (Gas6) were also identified but at the same levels in SLE- and HC-MPs. The anionic phospholipid-binding plasma protein β_2_-glycoprotein I was significantly decreased in SLE-MP but not identified in the SSc–HC sample set. The type I interferon-inducible G3BP which previously was found to be highly abundant on SLE-MPs [19, 34] was also increased here (cf. Fig. 2).

Mediators and effectors of apoptosis: Caspase-3, BH3-interacting domain death agonist (*BID*), cytochrome c, calpains, and PDCD6 interacting protein (*PDCD6IP*, Alix) were all significantly increased in SLE-MPs (Fig. 5b). These proteins were all unchanged in SSc versus HC-MPs. Caspase-3 was present at low levels (undetectable in 20% of the SLE samples) but still clearly increased compared with HC samples (*q* = 0.0071) (Fig. 5b), and caspase-3 and cytochrome c levels correlated (*p* = 0.007) in SLE-MPs (Additional file 1: Fig. S7). No other caspases were detected. The granzyme A-activated DNAse (*NME1*) which induces a caspase-3 independent, *TREX1*-dependent, cell death pathway [35] was highly increased (*q* = 2.9 × 10^−8^) in SLE-MPs (Fig. 5c). This was also the case for the apoptosis-modulating proteins [36, 37] β-arrestin 1 (*q* < 0.0001) and tumor necrosis factor α-induced protein 8 (*TNFAIP8*) (*q* = 0.0012). Histones H2A, H2B, H3, and H4 were detected at variable but low intensities (abundance rank around 700/1139) and were found in HC, SSc, and SLE samples at similar levels.

As for proteins maintaining phospholipid bilayer asymmetry, cell cycle control protein 50A (*TMEM30A*) and anoctamin-6 (*TMEM16F*), an ATP-dependent flippase and a scramblase, respectively, were both significantly decreased in SLE-MPs (Additional file 1: Fig. S8) and not altered in SSc-MP.

### Metabolic and oxidative defense status of SLE-MPs

**Fig. 6:**
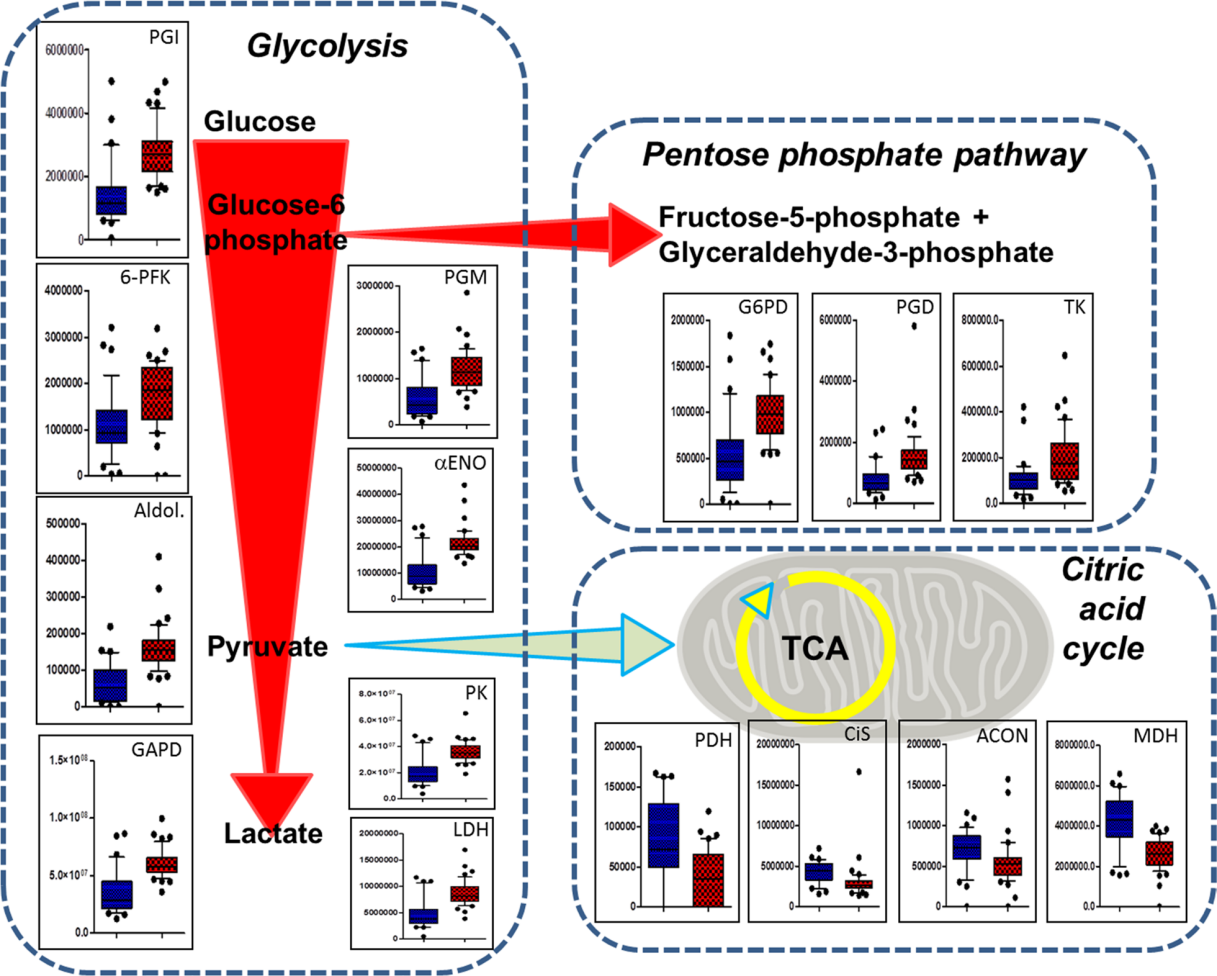
Specific metabolic profile of SLE-MPs. Simplified diagrams of pathways with *box plots* (medians and interquartile ranges, *whiskers* at 10–90 percentiles; *red* SLE; *blue* HC) of the intensities of 15 specific enzymes inserted. PGI, phosphoglucose isomerase; 6-PFK, 6-phosphofructokinase; Aldol., transaldolase; GAPD, glyceraldehyde-3-phosphate dehydrogenase; PGM, Phosphoglycerate mutase; αENO, alpha-enolase; PK, Pyruvate kinase; LDH, lactate dehydrogenase; PDH, pyruvate dehydrogenase; CiS, citrate synthase; ACON, aconitase; MDH, Malate dehydrogenase; G6PD, Glucose-6-phosphate dehydrogenase; PGD, 6-phosphogluconate dehydrogenase; TK, transketolase. All differences between SLE and HC are highly significant (*q* < 0.002, Mann–Whitney, two-tailed, Benjamini–Hochberg adjusted)

Glycolytic and pentose phosphate pathway enzymes were strikingly, significantly, and uniformly upregulated in SLE-MPs. In contrast, all identified citric acid cycle enzymes were significantly down-regulated (Fig. 6). The eukaryotic initiation factor 4A-II (*EIF4A2*) was highly significantly (*q* < 0.0002) increased in the SLE-MPs (and not in SSc-MP). Its increase in SLE-MPs might reflect increased protein synthesis in parent cells. Additional indications of metabolically highly active and stressed progenitors of SLE-MPs were increases in non-mitochondrial heat shock proteins—including HSP90β, HSP90α, HSP73, HSP70 (Additional file 1: Fig. S9), and HSC70. This was not observed among the SSc-MPs.

Alterations in oxidative defense proteins showed a complicated pattern which might reflect increased oxidative stress in a specific cellular context. Thus, enzymatic antioxidant scavengers superoxide dismutase 1, thioredoxin, and peroxiredoxins 1 and 6 were significantly increased in SLE-MPs while others such as peroxiredoxins 3 (mitochondrial) and 4 (endoplasmatic reticulum), glutathione peroxidase 3 (*GPX3*), a plasma protein, and manganese superoxide dismutase (mitochondrial) were significantly decreased. Catalase, peroxiredoxins 2 and 5 (mitochondrial), and glutathione peroxidase 1 were unchanged. Other oxidative stress-related and inflammation-induced proteins such as 4 of 5 identified protein disulfide isomerases were significantly upregulated in SLE-MP but unchanged in SSc-MP. Finally, chloride intracellular channel protein 1 (*CLIC1*), which is an oxidation sensor was highly increased in SLE-MPs (*q* = 2.9 × 10^−8^).

Proteasome activator 28 (PA28) α and β subunits were both significantly upregulated in SLE-MPs and were strongly correlated (*p* < 0.0001) in the individual samples (Additional file 1: Fig. S9). No MHC class II molecules were identified in any samples but 26 individual MHC class I molecules were found of which 5 were significantly decreased and one (HLA-Bw50) significantly elevated (data not shown). No significant differences were found in the HC-SSc data set regarding 22 identified MHC class I molecules.

### Proteins of intracellular signaling

The Ras-related small guanosine triphosphate (GTP)-binding proteins (GTPases) are involved in cell growth, cytoskeletal organization, and secretion. The Rab proteins comprise the largest family within the Ras superfamily. Rho and Arf proteins are other subfamilies. A total of 14 Rab proteins and 2 Rab GDP dissociation inhibitors (GDI) were identified and showed a diverse behavior (Additional file 1: Fig. S10). Thus, Rab-8A/B and GDI1/2 were significantly and specifically increased in SLE-MP while the remaining Rab proteins were either decreased (most pronounced for Rab-37) or not significantly different. All identified Rho family proteins (including RhoA, cdc42, Rac-1, Rac-2) (Additional file 1: Fig. S10B) and Arf1/3 were significantly increased. Another GTPase, dynamin-1 like protein, was highly increased in SLE-MPs (*q* = 4 × 10^−5^)(data not shown). The same was true for yet another intracellular signaling and cytoskeletal regulator, pleckstrin (*q* < 0.0001).

The Bruton tyrosine kinase (BTK), related to the *Src* family of cytoplasmic tyrosine kinases, was almost twofold increased in SLE MPs (*q* < 0.0001). It was not statistically significantly altered in the SSc-MP proteome (Additional file 1: Fig. S11). Also, *Src1* tyrosine kinase and protein tyrosine phosphatase, non-receptor type 6 (*PTNP6*), were significantly upregulated in SLE-MPs (*q* < 3 × 10^−7^).

## Discussion

In our cohort of SLE, SSc, and HC-MP samples, the SLE-MP proteomes proved to be very distinct and we therefore hypothesize the existence of an MP population—which we call luposomes—that is specific for SLE patients. Apart from immunoglobulins and complement proteins—that are also associated with SSc-MPs—the luposomes are distinguished by lacking mitochondrial and platelet proteins and by having profound cytoskeletal alterations including losses of myosin heavy and light chains and increased actin-interacting proteins. *MYH9* is an apoptotic caspase substrate [38], and SNPs in the *MYH9* gene are associated with increased incidence of lupus nephritis in European-Americans [39]. Myosin light chain kinase (*MLCK*), which is strongly increased in SLE-MPs, is involved in nuclear shrinkage and loading of nuclear material into apoptotic bodies [40]. Luposomes are also distinguished by highly increased glycolytic and pentose phosphate shunt enzyme levels and increased levels of various small GTPases, tyrosine kinases, and phosphatases. Metabolic studies of SLE T-lymphocytes and neutrophils show decreased glucose uptake, increased aerobic glycolysis, decreased ATP, and an increase of basal lactate levels and cell apoptosis [41]. These metabolic aberrations may reflect mitochondrial deficiencies because of activation-induced cell death and chronic antigen stimulation. The diminished abundance of mitochondrial proteins in SLE-MPs is compatible with apoptosis-related mechanisms of mitochondrial dissolution, but could also be due to an increased number of MPs from cell types with relatively few mitochondria such as neutrophils and lymphocytes. Likewise, while the increased abundance of glycolytic enzymes agrees [33] with an apoptotic origin of SLE particles, an increased MP production from metabolically reprogrammed cells, i.e., cells relying on glycolysis for energy production (the Warburg effect [42]), may also contribute.

Luposomes do not classify as exosomes since they are larger and there is no increase of markers [43] such as flotillins, vacuolar sorting proteins, integrins, tetraspanins, and annexin A2 in the SLE-MPs. An exception is the programmed cell death 6 interacting protein (*ALIX*) which is markedly increased. However, this protein is not exosome-specific [43]. Further, luposomes are not conventional cell membrane-derived MPs (also called ectosomes) which contain mitochondrial, ribosomal, nucleolar, and centrosomal proteins [43]. SLE-MPs do display high levels of some of the putative ectosome-specific proteins such as *TKT*, Rab8B, and *GSTP1* and the recently proposed marker of medium sized EVs, actinin-4, but other markers, such as mitofilin and GP96, are drastically decreased and unaltered, respectively [32, 43]. In contrast, in addition to the cytoskeletal aberrations, increased *MLCK,* and increased glycolytic enzymes, the increased cytochrome c, caspase 3, BID, calpain 1, and *ALIX* as well as increased calreticulin (*CRT*) are all in agreement with an apoptotic origin of immunogenic luposomes. Since histones are often used to indicate apoptotic rather than cell membrane origins of MPs [44], the unremarkable differences in histone content and the absence of the high-mobility group protein 1 (*HMGB1*) seem to disagree with this notion. However, not all studies find that histones are enriched on apoptotic vesicles [33]. Also, protein identification in the present study depends on trypsin-mediated protein fragmentation, which may be inhibited in proteins complexed—and thus protected against proteolysis—with autoantibodies, such as histones in self-antigens represented by MP-associated chromatin [11]. Finally, we did not analyze for acetylated, hypercitrullinated [45], or methylated histone variants [18]. As for the cellular origin of SLE-MPs it is interesting that BTK, a molecule normally mostly expressed in B-cells is heavily upregulated in SLE-MPs. BTK has roles in apoptotic cell uptake [46] and is a key molecule for TLR9 signaling in plasmacytoid dendritic cells [47]. Even though transcripts in non-B-cells are normally down-regulated, BTK may be expressed in all hematopoietic cell types including myeloid lineages [48] and thus its increased abundance in SLE-MPs cannot be concluded to be due to increased B-cell MP formation.

The presence of the Ca(II)-dependent phosphoserine-binding annexin V as an MP-associated protein was surprising since annexin V is not expected to be surface-bound to MPs from citrate plasma. Thus, the significantly increased annexin V associated with SLE-MPs must bind Ca^2+^-independently and/or be present inside MPs. Increased Ca^2+^-independent surface-bound endogenous annexin V may explain the decreased number of MPs binding exogeneously added annexin V in SLE samples [17]. A pro-clearance profile on SLE-MPs is also consistent with the decreased level of phagocytosis-inhibiting CD47. Further, since CD47 restricts dendritic cell (DC) signaling, decreased CD47 in antigenic material (such as SLE-MPs) would increase e.g. splenic DC or plasmacytoid DC activation [49].

Regarding the cellular provenance of luposomes, platelets are unlikely because both flow cytometry and proteomics show the paucity of normal PMPs below 1 μm in the SLE-MP population. An increase of larger (apoptotic) particles in the SLE samples suppressing the relative PMP abundance cannot be ruled out, but by EM we found no overt size-distribution differences between SLE- and HC-MP samples, and the findings of lowered total numbers of MP by flow cytometry persisted also when using a gate limit of 2 μm [17]. Further, we find no evidence, despite recent reports [18], of increased endothelial cell-derived MPs. Instead, CD31 (PECAM-1) is significantly decreased. In contrast to the strongly decreased platelet and endothelial cell markers there were no significant differences regarding neutrophil markers such as CD11b and the possible relationship of SLE-MPs to lupus low-density granulocytes and NETosis [50] should be explored. Thus, except for clear indications of decreased numbers of conventional PMPs suggesting increased clearance of normal MPs in SLE, no classical blood cell or monospecific endothelial cell surface marker was significantly decreased or increased in the SLE-MP proteome.

In short, luposomes lack mitochondria, contain a disrupted cytoskeleton, and have a very high abundance of glycolytic enzymes. The observed luposome proteome is the result of an intricate combination of differentially regulated proteins in SLE, contributions of different parent cells, and differences in the processes generating and removing MPs. It is not readily possible using the present data to discriminate between the relative impact of these factors. The data from the SSc-MPs, i.e., from another systemic immunoinflammatory disease, strongly suggest that the SLE-MPs are not of a type arising with chronic inflammation as such, but that they are an SLE-specific phenomenon. Further investigations of new sample sets using e.g. specific immuno-isolation in combination with proteomic methods will be necessary to elucidate luposome-generating mechanisms. The present data do, however, indicate that luposomes are attractive objects for refined diagnostics and for further unravelling and counteracting the molecular pathology associated with SLE autoimmunity.

## Additional files

**Additional file 1.** Supplementary Information (**Table S1**, **Figures S1–S11**).

**Additional file 2.** Data file SLE-HC.xlsx.

**Additional file 3.** Data file SSc-HC.xlsx.

## Abbreviations

APS: antiphospholipid syndrome
AUC: area under the curve
BTK: Bruton’s tyrosine kinase
C: complement protein(s)
ER: endoplasmic reticulum
EV: extracellular vesicles
FGA, FGB, FGG: fibrinogen α, β, and γ chains, respectively
G3BP: galectin-3 binding protein
GPX3: glutathione peroxidase 3
HC: healthy control
HPX: hemopexin
HSP: heat shock protein
IBAQ: intensity-based absolute quantification
Ig: immunoglobulin
MIF: macrophage migration inhibitory factor
MP: microparticle
MS: mass spectrometry
MYH9: myosin-9
ORM: orosomucoid
PPP: platelet-poor plasma
ROC: receiver operating characteristics
SAA: serum amyloid A
SLE: systemic lupus erythematosus
SLEDAI: SLE disease activity index
SSc: systemic sclerosis

## References

[1] Magna M, Pisetsky DS (2015). The role of cell death in the pathogenesis of SLE: is pyroptosis the missing link?. Scand J Immunol.

[2] Yanez-Mo M, Siljander PR, Andreu Z, Zavec AB, Borras FE, Buzas EI (2015). Biological properties of extracellular vesicles and their physiological functions. J Extracell Vesicles.

[3] Buzas EI, Gyorgy B, Nagy G, Falus A, Gay S (2014). Emerging role of extracellular vesicles in inflammatory diseases. Nat Rev Rheumatol.

[4] Crow MK, Type I (2014). Interferon in the pathogenesis of lupus. J Immunol.

[5] Robey FA, Jones KD, Steinberg AD (1985). C-reactive protein mediates the solubilization of nuclear DNA by complement in vitro. J Exp Med.

[6] Herrmann M, Voll RE, Zoller OM, Hagenhofer M, Ponner BB, Kalden JR (1998). Impaired phagocytosis of apoptotic cell material by monocyte-derived macrophages from patients with systemic lupus erythematosus. Arthritis Rheum.

[7] Pickering MC, Botto M, Taylor PR, Lachmann PJ, Walport MJ (2000). Systemic lupus erythematosus, complement deficiency, and apoptosis. Adv Immunol.

[8] Walport MJ (2000). Lupus, DNase and defective disposal of cellular debris. Nat Genet.

[9] Pisetsky DS, Gauley J, Ullal AJ (2011). HMGB1 and microparticles as mediators of the immune response to cell death. Antioxid Redox Signal.

[10] Biermann MH, Veissi S, Maueroder C, Chaurio R, Berens C, Herrmann M (2014). The role of dead cell clearance in the etiology and pathogenesis of systemic lupus erythematosus: dendritic cells as potential targets. Expert Rev Clin Immunol.

[11] Sisirak V, Sally B, D’Agati V, Martinez-Ortiz W, Ozcakar ZB, David J (2016). Digestion of chromatin in apoptotic cell microparticles prevents autoimmunity. Cell.

[12] Colonna L, Lood C, Elkon KB (2014). Beyond apoptosis in lupus. Curr Opin Rheumatol.

[13] Flaumenhaft R, Dilks JR, Richardson J, Alden E, Patel-Hett SR, Battinelli E (2009). Megakaryocyte-derived microparticles: direct visualization and distinction from platelet-derived microparticles. Blood.

[14] Smalley DM, Root KE, Cho H, Ross MM, Ley K (2007). Proteomic discovery of 21 proteins expressed in human plasma-derived but not platelet-derived microparticles. Thromb Haemost.

[15] Ostergaard O, Nielsen CT, Iversen LV, Jacobsen S, Tanassi JT, Heegaard NH (2012). Quantitative proteome profiling of normal human circulating microparticles. J Proteome Res.

[16] Ullal AJ, Reich CF, Clowse M, Criscione-Schreiber LG, Tochacek M, Monestier M (2011). Microparticles as antigenic targets of antibodies to DNA and nucleosomes in systemic lupus erythematosus. J Autoimmun.

[17] Nielsen CT, Ostergaard O, Johnsen C, Jacobsen S, Heegaard NH (2011). Distinct features of circulating microparticles and their relationship to clinical manifestations in systemic lupus erythematosus. Arthritis Rheum.

[18] Dieker J, Tel J, Pieterse E, Thielen A, Rother N, Bakker M (2016). Circulating apoptotic microparticles in systemic lupus erythematosus patients drive the activation of dendritic cell subsets and prime neutrophils for NETosis. Arthritis Rheumatol.

[19] Ostergaard O, Nielsen CT, Iversen LV, Tanassi JT, Knudsen S, Jacobsen S (2013). Unique protein signature of circulating microparticles in systemic lupus erythematosus. Arthritis Rheum.

[20] Hochberg MC (1997). Updating the American College of Rheumatology revised criteria for the classification of systemic lupus erythematosus. Arthritis Rheum.

[21] Miyakis S, Lockshin MD, Atsumi T, Branch DW, Brey RL, Cervera R (2006). International consensus statement on an update of the classification criteria for definite antiphospholipid syndrome (APS). J Thromb Haemost.

[22] Preliminary criteria for the classification of systemic sclerosis (scleroderma). Subcommittee for scleroderma criteria of the American Rheumatism Association Diagnostic and Therapeutic Criteria Committee. Arthritis Rheum 1980;23:581–90. 10.1002/art.1780230510 7378088

[23] LeRoy EC, Black C, Fleischmajer R, Jablonska S, Krieg T, Medsger TA (1988). Scleroderma (systemic sclerosis): classification, subsets and pathogenesis. J Rheumatol.

[24] Clements P, Lachenbruch P, Siebold J, White B, Weiner S, Martin R (1995). Inter and intraobserver variability of total skin thickness score (modified Rodnan TSS) in systemic sclerosis. J Rheumatol.

[25] Petri M, Buyon J, Kim M (1999). Classification and definition of major flares in SLE clinical trials. Lupus.

[26] Rappsilber J, Ishihama Y, Mann M (2003). Stop and go extraction tips for matrix-assisted laser desorption/ionization, nanoelectrospray, and LC/MS sample pretreatment in proteomics. Anal Chem.

[27] Schwanhausser B, Busse D, Li N, Dittmar G, Schuchhardt J, Wolf J (2011). Global quantification of mammalian gene expression control. Nature.

[28] Benjamini Y, Hochberg Y (1995). Controlling the false discovery rate: a practical and powerful approach to multiple testing. J R Stat Soc Ser B.

[29] Braga-Lagache S, Buchs N, Iacovache MI, Zuber B, Jackson CB, Heller M (2016). Robust label-free, quantitative profiling of circulating plasma microparticle (MP) associated proteins. Mol Cell Proteomics.

[30] Nielsen CT, Ostergaard O, Stener L, Iversen LV, Truedsson L, Gullstrand B (2012). Increased IgG on cell-derived plasma microparticles in systemic lupus erythematosus is associated with autoantibodies and complement activation. Arthritis Rheum.

[31] Crescitelli R, Lasser C, Szabo TG, Kittel A, Eldh M, Dianzani I (2013). Distinct RNA profiles in subpopulations of extracellular vesicles: apoptotic bodies, microvesicles and exosomes. J Extracell Vesicles.

[32] Kowal J, Arras G, Colombo M, Jouve M, Morath JP, Primdal-Bengtson B (2016). Proteomic comparison defines novel markers to characterize heterogeneous populations of extracellular vesicle subtypes. Proc Natl Acad Sci USA.

[33] Ucker DS, Jain MR, Pattabiraman G, Palasiewicz K, Birge RB, Li H (2012). Externalized glycolytic enzymes are novel, conserved, and early biomarkers of apoptosis. J Biol Chem.

[34] Nielsen CT, Ostergaard O, Rekvig OP, Sturfelt G, Jacobsen S, Heegaard NH (2015). Galectin-3 binding protein links circulating microparticles with electron dense glomerular deposits in lupus nephritis. Lupus.

[35] Lieberman J (2010). Granzyme A activates another way to die. Immunol Rev.

[36] Tan S, Li L, Chen T, Chen X, Tao L, Lin X (2015). beta-Arrestin-1 protects against endoplasmic reticulum stress/p53-upregulated modulator of apoptosis-mediated apoptosis via repressing p-p65/inducible nitric oxide synthase in portal hypertensive gastropathy. Free Radic Biol Med.

[37] Porturas TP, Sun H, Buchlis G, Lou Y, Liang X, Cathopoulis T (2015). Crucial roles of TNFAIP8 protein in regulating apoptosis and Listeria infection. J Immunol.

[38] Simon GM, Dix MM, Cravatt BF (2009). Comparative assessment of large-scale proteomic studies of apoptotic proteolysis. ACS Chem Biol.

[39] Lin CP, Adrianto I, Lessard CJ, Kelly JA, Kaufman KM, Guthridge JM (2012). Role of MYH9 and APOL1 in African and non-African populations with lupus nephritis. Genes Immun.

[40] Zirngibl M, Furnrohr BG, Janko C, Munoz LE, Voll RE, Gregory CD (2015). Loading of nuclear autoantigens prototypically recognized by systemic lupus erythematosus sera into late apoptotic vesicles requires intact microtubules and myosin light chain kinase activity. Clin Exp Immunol.

[41] Li KJ, Wu CH, Hsieh SC, Lu MC, Tsai CY, Yu CL (2012). Deranged bioenergetics and defective redox capacity in T lymphocytes and neutrophils are related to cellular dysfunction and increased oxidative stress in patients with active systemic lupus erythematosus. Clin Dev Immunol.

[42] Darekar S, Georgiou K, Yurchenko M, Yenamandra SP, Chachami G, Simos G (2012). Epstein-Barr virus immortalization of human B-cells leads to stabilization of hypoxia-induced factor 1 alpha, congruent with the Warburg effect. PLoS ONE.

[43] Keerthikumar S, Gangoda L, Liem M, Fonseka P, Atukorala I, Ozcitti C (2015). Proteogenomic analysis reveals exosomes are more oncogenic than ectosomes. Oncotarget.

[44] Radic M, Marion T, Monestier M (2004). Nucleosomes are exposed at the cell surface in apoptosis. J Immunol.

[45] Wang Y, Li M, Stadler S, Correll S, Li P, Wang D (2009). Histone hypercitrullination mediates chromatin decondensation and neutrophil extracellular trap formation. J Cell Biol.

[46] Byrne JC, Ni GJ, Stacey KB, Coffey BM, McCarthy E, Thomas W (2013). Bruton’s tyrosine kinase is required for apoptotic cell uptake via regulating the phosphorylation and localization of calreticulin. J Immunol.

[47] Wang J, Lau KY, Jung J, Ravindran P, Barrat FJ (2014). Bruton’s tyrosine kinase regulates TLR9 but not TLR7 signaling in human plasmacytoid dendritic cells. Eur J Immunol.

[48] Smith CI, Baskin B, Humire-Greiff P, Zhou JN, Olsson PG, Maniar HS (1994). Expression of Bruton’s agammaglobulinemia tyrosine kinase gene, BTK, is selectively down-regulated in T lymphocytes and plasma cells. J Immunol.

[49] Yi T, Li J, Chen H, Wu J, An J, Xu Y (2015). Splenic dendritic cells survey red blood cells for missing self-CD47 to trigger adaptive immune responses. Immunity.

[50] Lood C, Blanco LP, Purmalek MM, Carmona-Rivera C, De Ravin SS, Smith CK (2016). Neutrophil extracellular traps enriched in oxidized mitochondrial DNA are interferogenic and contribute to lupus-like disease. Nat Med.

