# Supplementary material for: Distinct proteome pathology of circulating microparticles in systemic lupus erythematosus
Source: Clin Proteomics. 2017 Jun 21;14:23. doi: 10.1186/s12014-017-9159-8 (PMC5479039; doi:10.1186/s12014-017-9159-8)
Supplement: Supplementary file 1 — Additional file 1. Supplementary Information (Table S1, Figures S1–S11). [file 12014_2017_9159_MOESM1_ESM.docx]

Distinct Proteome Pathology of Circulating Microparticles in Systemic Lupus Erythematosus

**Supplementary Material**

Table S1. Systemic lupus erythematosus patient characteristics at inclusion. (Page 2)

Figure S1. Correlation of β-actin abundance with Intracellular cytoskeletal and filamentous proteins. (Page 3)

Figure S2. Significantly altered abundance of actin-binding proteins in SLE-MPs. (Page 4)

Figure S3. Acute phase proteins in the SLE-HC and the SSc-HC MP samples. (Page 5)

Figure S4. Unsupervised hierarchical clustering of 930 MP-proteins based on normalized

intensities.

Figure S5. Cell-specific and surface CD molecules in SLE and HC-MPs. (Page 6)

Figure S6. Flotillins-1 and -2. (Page 7)

Figure S7. Correlation between cytochrome c and caspase-3 abundance in SLE MP samples. (Page 8)

Figure S8. Scramblase and flippase in SLE-MPs. (Page 9)

Figure S9. Increased heat shock and proteasome proteins in SLE-MPs. (Page 10)

Figure S10. Intracellular signaling and cytoskeleton-regulating small GTP proteins. (Page 11)

Figure S11. Increased Bruton tyrosine kinase in SLE-MPs. (Page 12)

SUPPLEMENTARY TABLE S1

| Systemic lupus erythematosus patient characteristics at inclusion* | |
| --- | --- |
| Number**, female/male | 42/3 |
| Age, years | 40 (22-76) |
| Disease duration, years** | 9 (0-24) |
| Disease manifestations at inclusion** |  |
| Nephritis | 10 (22) |
| Vasculitis | 4 (9) |
| Arthritis | 4 (9) |
| Rash | 2 (4) |
| Anti-phospholipid syndrome | 13 (29) |
| SLEDAI** | 4 (0-21) |
| SLICC Damage Index** | 0 (0-8) |
| Medication** |  |
| Prednisolone | 19 (43) |
| Prednisolone dose, mg | 0 (0-80) |
| Anti-malarials | 9 (20) |
| Methotrexate, azathioprine or mycophenylate  mofetil | 19 (43) |
| Biochemistry** |  |
| Hemoglobin (mM) | 7,8 (4,6-10,5) |
| Leukocytes (10^9^/L) | 5,6 (2,2-12,0) |
| Lymphocytes (10^9^/L) | 1,0 (0,2-3,2) |
| Platelets (10^9^/L) | 237 (45-505) |
| Serology and complement activation |  |
| ANA positive | 33 (73) |
| Anti-dsDNA | 17 (38) |
| Anti-Sm** | 2 (5) |
| Anti-Ro52 | 12 (27) |
| Anti-Ro60 | 10 (22) |
| Anti-La | 5 (11) |
| Anti-C1q | 10 (22) |
| Anti-cardiolipin IgG | 6 (13) |
| Anti-cardiolipin IgM | 5 (11) |
| Anti-β2-glycoprotein-I IgG | 3 (7) |
| Anti-β2-glycoprotein-I IgM | 1 (2) |
| Low C3 or C4 | 32 (71) |
| *Variables are presented either with numbers (percent) or median (range)  **One patient could only be classified with anti-phospholipid syndrome. Thus, only 44 patients are included in the calculations of the marked variables.  SLE=systemic lupus erythematosus; SLEDAI=systemic lupus erythematosus disease activity index; SLICC/DI=The Systemic Lupus International Collaborating Clinics Damage Index. | |

**Figure S1.** Correlation of β-actin abundance with Intracellular cytoskeletal and filamentous proteins. The correlation of β-actin abundance (raw intensity values) with the abundance of cytoskeletal and filamentous proteins in SLE-MPs (red) and HC-MPs (blue). Correlation coefficients and slopes for the regression lines fitted to the SLE and HC data are given (except for the spectrin plot). An altered ratio is seen for all myosins in the SLE-MPs, *i.e*., the slope of the lines for myosins (6 upper graphs, red regression lines) are approximately twice of the same regression lines in the HC samples. Protein names and UniProt identifiers stated on the X-axes.

**Figure S2.** Significantly altered abundance of actin-binding proteins in SLE-MPs. Plots comparing the abundance of actin-binding proteins in HC (blue) and SLE (red) MP samples. All depicted proteins are significantly increased in SLE-MPs (all *q* < 0.00025, Mann-Whitney, two-tailed). Horizontal lines mark medians.

**Figure S3.** Acute phase proteins in the SLE-HC and the SSc-HC MP samples. Ratios between intensity in disease samples and healthy samples are shown for the SLE-HC (orange) and the SSc-HC (green) sample sets for acute-phase and interferon type 1-induced proteins. G3BP, galectin-3-binding protein; SAA, serum amyloid A protein; ORM, orosomucoid; HPX, hemopexin; FG, fibrinogen (α, β, γ-chains).


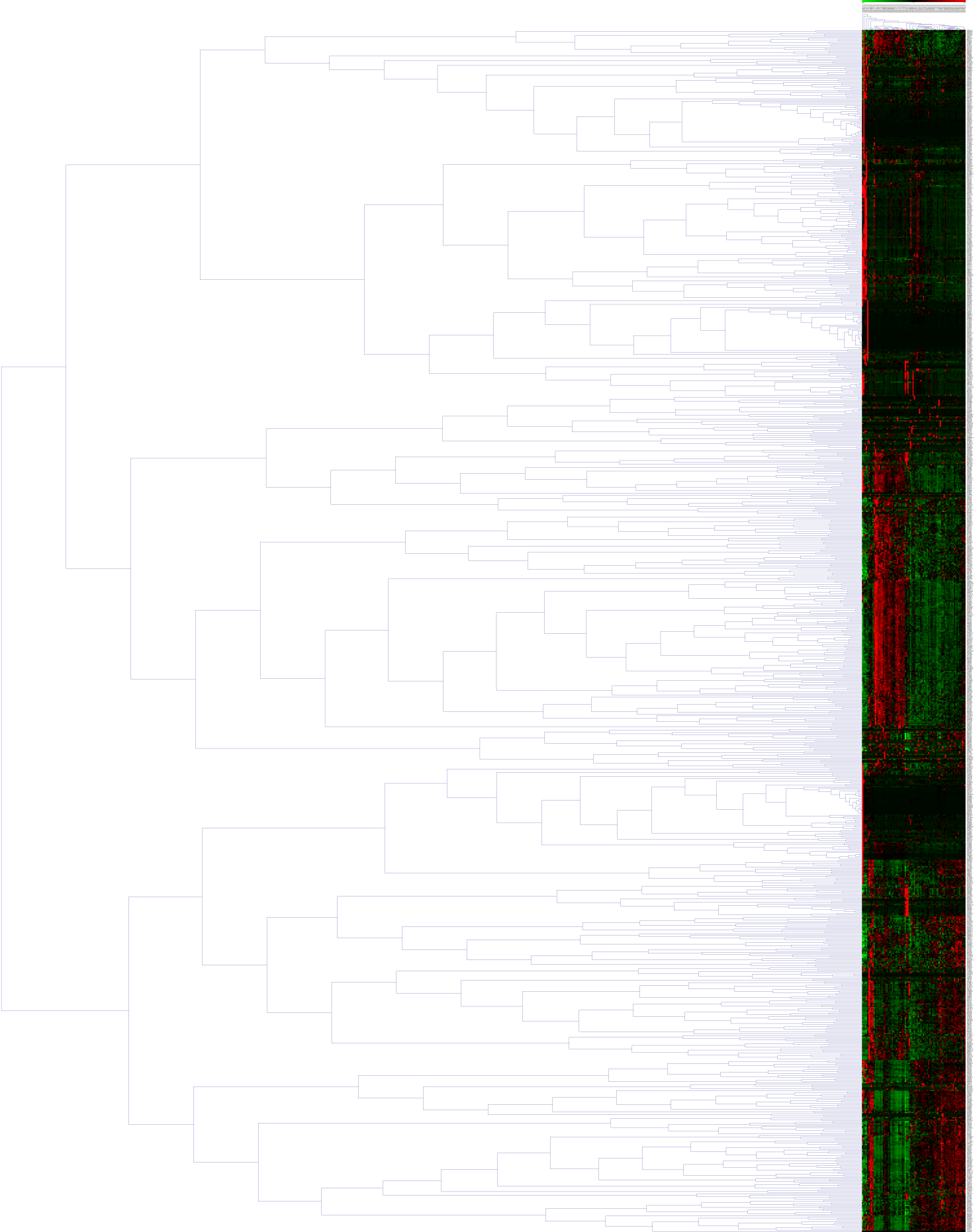


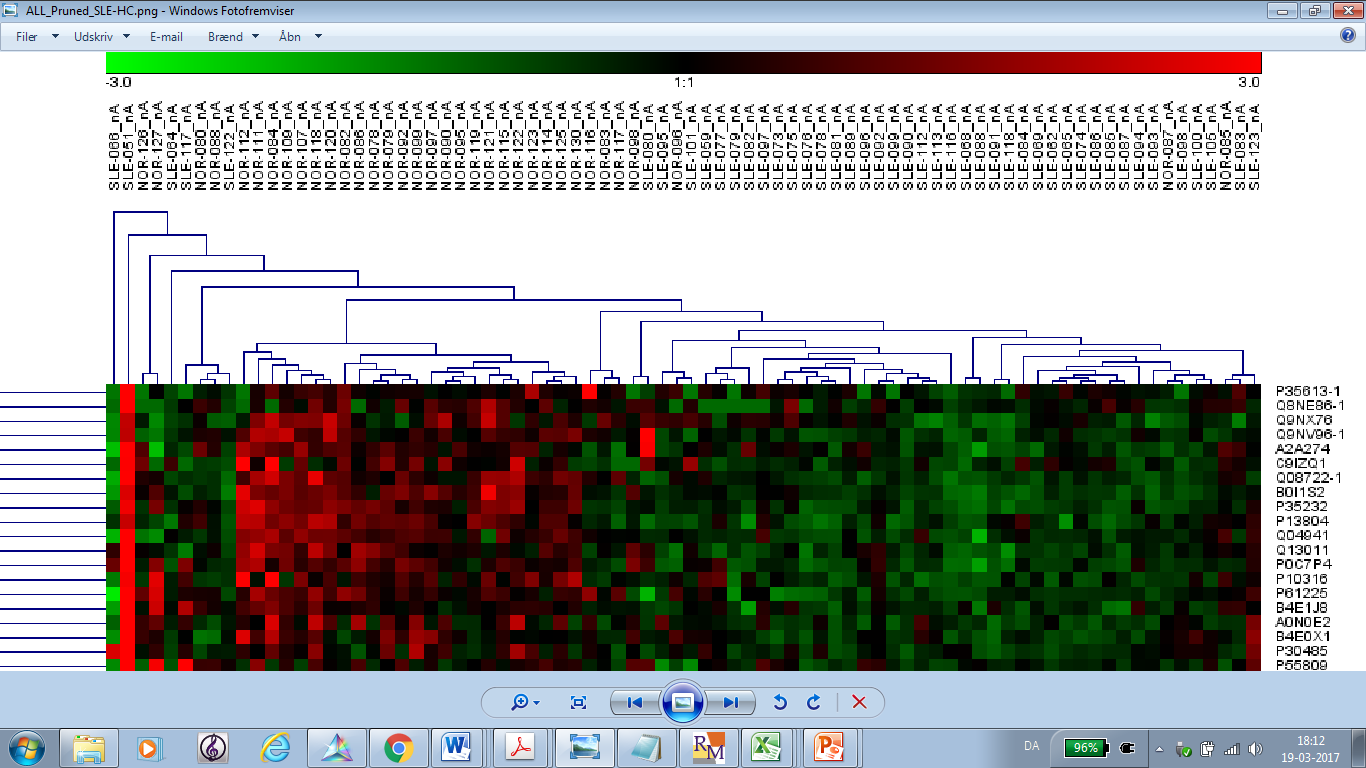


**Figure S4.** Unsupervised hierarchical clustering of 930 MP-proteins based on normalized intensities. The proteins represent the entire data set without immunoglobulins, complement proteins, hypothetical proteins and *Bos taurus* proteins. To the right is shown an enlarged portion
of the horizontal axis (sample categories) illustrating the clustering of SLE and HC (NOR) samples.

**Figure S5.** Cell-specific and surface CD molecules in SLE and HC-MPs. Intensities of specific CD molecules in the two sample sets (HC, blue; SLE, red). CD names and UniProt designations are shown. Horizontal lines indicate medians. Differences between groups are non-significant (Mann-Whitney, two-tailed) when no *p*-values are shown.

**Figure S6.** Flotillins-1 and -2. Plots comparing the abundance of flotillin-1 and -2 in HC (blue) and SLE (red) MP samples. Both are significantly decreased in SLE-MPs (*p*-values indicated, Mann-Whitney, two-tailed). Horizontal lines mark medians and UniProt identifiers are included in the graph**.**

**Figure S7.** Correlation between cytochrome c and caspase-3 abundance in SLE MP samples. Intensity values of cytochrome c (CytC) as a function of intensity values of caspase-3 (CASP3) in SLE-MPs. Both proteins are significantly increased in SLE-MPs compared to healthy controls (*cf.* **Fig. 5** in the main document) although caspase-3 is not detected in 20% of the SLE samples. There is a significant correlation using Spearman’s test as indicated on the figure.

**Figure S8.** Scramblase and flippase in SLE-MPs. Plots comparing the abundance of a scramblase and flippase in HC (blue) and SLE (red) MP samples. Both are significantly decreased in SLE-MPs (p-values indicated, Mann-Whitney, two-tailed). Horizontal lines mark medians and UniProt identifiers are included in the graph.


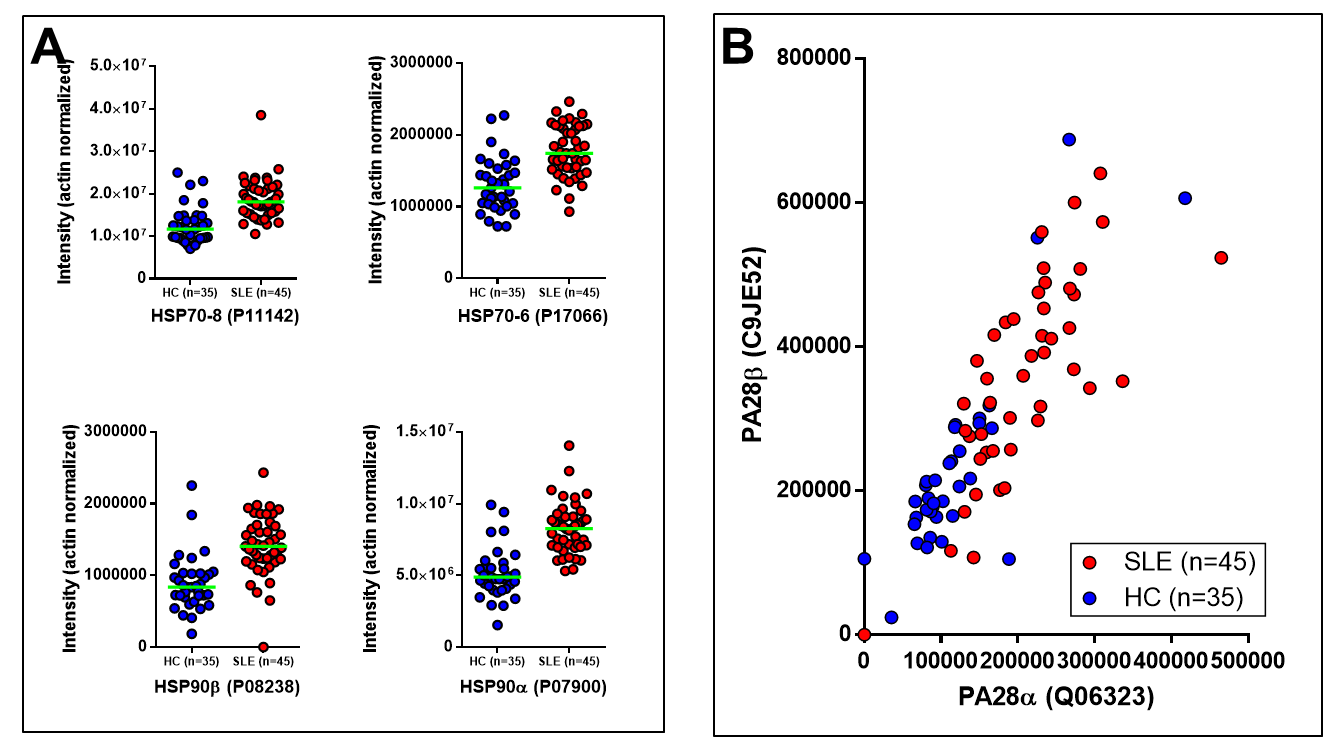


**Figure S9.** Increased heat shock and proteasome proteins in SLE-MPs. **A,** Levels of different heat shock proteins in HC (blue) and SLE (red) MPs. All are highly significantly increased (*q* < 0.0001) in SLE-MPs (Mann-Whitney, two-tailed, Benjamini-Hochberg adjusted). **B,** Correlation between the proteasomal PA28 α and β subunits - both significantly increased in SLE-MPs (*q* < 0.0001) - in the HC+SLE sample set (Spearman r = 0.85, *p* < 0.0001).

**
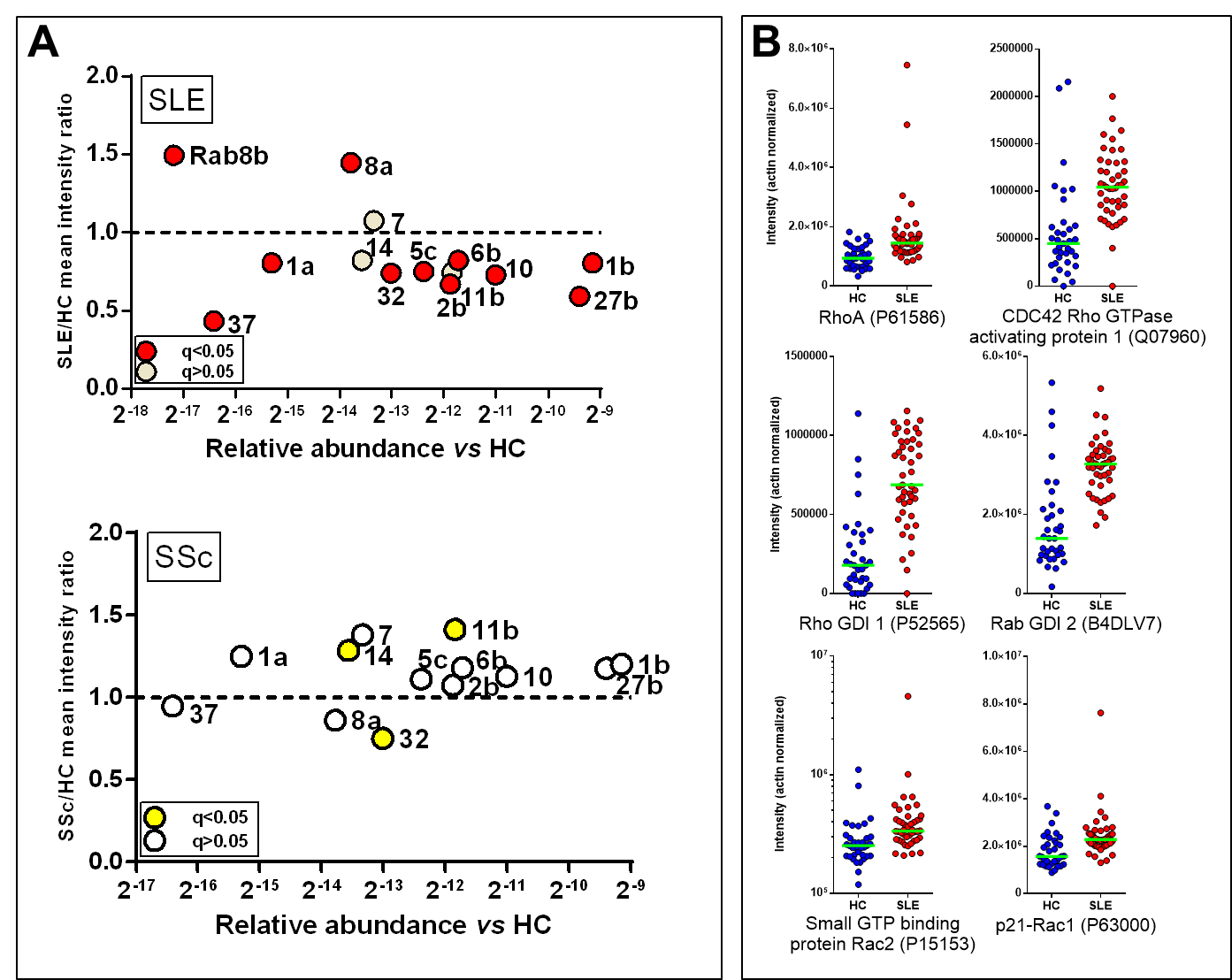
**

**Figure S10.** Intracellular signaling and cytoskeleton-regulating small GTP proteins. **A,** All identified Rab proteins in the SLE-HC (upper) and the SSc-HC (lower) sample sets are show with their mean intensity ratio (diseased *vs.* healthy) as a function of the intensity-based abundance (IBAQ) in the HC samples. The Rab proteins are identified by their numbers and by the significance of their increase/decrease in the disease samples (*q* < 0.05 are marked with bright red (SLE/HC) and bright yellow (SSc/HC), respectively). **B,** Levels of Rho and Rac family members and Rab GDP dissociation inhibitors (GDI) in SLE (red) and HC (blue) samples. All were highly significantly (*q* < 0.001) increased in SLE.

**Figure S11.** Increased Bruton tyrosine kinase (BTK) in SLE-MPs. The distribution of protein abundance in the two sample sets (SLE-HC, left and SSc-HC, right). There was no significant difference in the SSc-MPs *vs.* HC-MPs samples but a highly significant increase in the SLE-MPs *vs.* HC-MPs.
